# Fuscoside Attenuates Bone Loss in Bone Defects by Regulating The Rankl/Nlrp3/Opg Pathway in Rats

**DOI:** 10.22074/cellj.2021.7736

**Published:** 2021-08-29

**Authors:** Xiangwei Liu, Binfeng Wang

**Affiliations:** Department of Traumatic Orthopedics, Chifeng Municipal Hospital, Chifeng, Inner Mongolia, China

**Keywords:** Bone Defect, Cytokines, Fuscoside, Nlrp3, Osteoclast

## Abstract

**Objective:**

This study evaluated the beneficial effect of fuscoside in the repair of bone defects (BDs) and the possible molecular mechanism thereof.

**Materials and Methods:**

In this experimental study, a BD was induced by drilling the rat tibia. The rats were then administered oral fuscoside, at 200 or 300 mg/kg, for 2 weeks. The effect of treatment was assessed based on the bone formation score and on the levels of cytokines and biochemical markers in serum. Tibial expression of the proteins involved in the Rankl/Nlrp3/Opg pathway was determined by quantitative reverse-transcription polymerase chain reaction and western blot assay, and histopathological changes by haematoxylin and eosin and TRAP staining.

**Results:**

In the fuscoside-treated BD rats, the bone formation score improved and inflammatory cytokine levels were reduced. The levels of biochemical markers improved as well, as did the expression of apoptosis proteins. Fuscoside also attenuated the expression of Rankl, Opg, Nlrp3, Runx2, Osterix, and Osteocalcin (Oc) proteins in the tibial tissue of the BD rats and reversed the abnormal histopathological changes.

**Conclusion:**

These results suggest that fuscoside improves BD repair by reducing the differentiation of osteoclasts and by regulating the Rankl/Nlrp3/Opg pathway.

## Introduction

Bone fractures are common in adolescents and children, as the growth plate cartilage and weak areas of the long bones are prone to injury and fracture (1). A bone defect (BD) reflects the undesirable growth of bone tissue during the repair of a bone fracture or growth plate injury (2). Both the repair itself and defect formation involve many factors, including the extracellular matrix and the expression of a large number of genes from different cell types that interact both temporally and spatially. Fracture healing is complex and involves inflammation, intramembranous and endochondral ossification, and bone remodelling (3) in processes such as the differentiation and proliferation of bone cells, extracellular matrix deposition, gene expression and cell signalling (4). BDs arise from osteocyte activity, which is regulated by osteoclastogenesis-inhibitory factor, osteocalcin (Oc), osteoprotegerin (Opg), Runx-2, bone sialoprotein and Nlrp3 (5). For instance, overexpression of Nlrp3 was shown to delay wound healing, and its reduced expression improved bone repair (6). 

There is no clear treatment for BDs, and their management in patients with metabolic disorder is particularly challenging. The development of therapeutic agents from natural sources has gained increasing attention, including for many chronic disorders. Fuscoside is a diterpene arabinose glycoside produced by the Caribbean soft coral *Eunicea fusca* (7). Among its properties are a strong anti-inflammatory activity by regulating interleukin (IL) production (8). Orally administered fuscoside induces vasodilatation and reduces brain oedema, probably due to its antagonism of vasopressin V1 (9). Thus, this study evaluated the protective effect of fuscoside on BD development.

## Materials and Methods

### Animals

In this experimental study, male Wistar rats weighing 180-225 g were housed following animal care guidelines at a humidity of 60 ± 5% and a temperature of 24 ± 3˚C under a 12-hours light/dark cycle. All animal experiments were approved by the Animal Ethical Committee of Chifeng Municipal Hospital, China (IAEC/CMH/2019/18).

### Chemicals

Fuscoside was procured from MedChemExpress (Monmouth Junction, NJ, USA). Enzyme-linked immunosorbent assay (ELISA) kits were purchased from R&D Systems (Minneapolis, MN, USA), and the antibodies used in the western blot assay from Thermo Fisher Scientific (Waltham, MA, USA). 

### Experiments

A BD was induced in the rat tibia as previously reported (10). Briefly, the animals were anaesthetised with intraperitoneal ketamine (60 mg/kg) and xylazine hydrochloride (10 mg/ kg). The skin over the tibia was then shaved and painted with iodine. A 2-cm longitudinal incision was made along the anteromedial border of the proximal tibia and the patellar tendon insertion was released. Tissues around the tibia were protected using a haemostat. A 1.5-mm micromotor drill was used to make a 3-mm BD on the proximal tibia. Saline was regularly applied to the drilled area to avoid overheating. The skin and muscle were then closed using 4-0 nylon sutures. The operated animals were housed in separated cages under controlled conditions until they were able to resume normal activities.

Four experimental groups were defined, with eight rats in each group: a sham group; a control BD group, operated on as described above and treated with dimethyl sulphoxide vehicle for the 2 weeks of the experiment; and fuscoside 200 and 300 mg/kg groups, operated on as described above and then treated with 200 or 300 mg fuscoside/kg orally for the 2 weeks of the study.

### Evaluation of bone defect healing

BD healing was evaluated as previously reported. Computed tomography imaging was used to estimate the bone healing effect and scored as follows: 0, no bone formation; 1, bone formation covering ≤ 25% of the defect; 2, bone formation covering 25-50% of the defect; 3, bone formation covering 51-75% of the defect; 4, bone formation covering 76-99% of the defect; 5, bone formation covering 100% of the defect.

### Determination of biochemical parameters

Blood was drawn from the retro-orbital plexus of anaesthetised rats and centrifuged at 2000 rpm for 10 minutes to separate the serum. The serum levels of OC, C-telopeptide of type 1 collagen (CTX) and bone-specific alkaline phosphatase (BSAP) were measured using ELISA kits following the manufacturers’ instructions (R&D Systems, Minneapolis, MN, USA).

### Measurement of cytokine levels

The serum levels of the inflammatory mediators IL-1β, IL-6, IL-10 and nuclear factor kappa B (NF-κB) were determined by ELISA using commercial kits following the manufacturer’s instructions (R&D Systems) (11).

### Determination of Rankl, Opg, Nlrp3, Runx2, Osterix and Oc mRNA expression

The relative expression of Rankl, Opg, Nlrp3, Runx2, Osterix and Oc and β-actin mRNA was estimated using SYBR green-based quantitative reverse transcription-polymerase chain reaction (qRT-PCR) as per previously reported method (12). TaqMan MicroRNA assays were performed after the extraction of Total RNA using TRIzol reagent. Total RNA (2 µg, reaction volume: 20 µL) was synthesized to form cDNA using moloney murine leukaemia virus reverse transcriptase. RT 2 SYBR Green Master Mix was mixed with the primers used in the study and expression of gene were determined by a quantitative SYBR Green PCR assay. The relative expression of the target gene was estimated using the 2^−ΔΔCq^ method (Table S1, See Supplementary Online Information at www.celljournal.org).

### Western blotting

Caspase-1, Bcl-2, Bax, Rankl, Opg, Nlrp3 And Runx2 expression were assessed in isolated tibia tissues by western blotting (13). Tissue homogenates was prepared and BCA kit (BioRad Laboratories, Hercules, CA, USA) was used to determine the protein content. Sodium dodecyl sulphate polyacrylamide gel electrophoresis on a 10% polyacrylamide gel was used to separate the protein content of tissue and further transferred to a membrane, which, after blocking in 5% blocking reagent, was incubated overnight at 4˚C with primary antibodies targeting Caspase-1 (1:200), Bcl-2 (1:200), Bax (1:200), Rankl (1:200), Opg (1:200), Nlrp3 (1:200) and Runx2 (1:200). Membranes were subsequently incubated with secondary antibodies for 60 minutes at room temperature. After the final wash, signals were developed using a Western Lightning ECL kit (PerkinElmer, Waltham, MA, USA). The results were analysed using Gel-Pro Analyzer 4.0 software. Densitometric levels of target proteins were quantified and normalised to that of β-actin.

### Histopathological analysis

The tibia was isolated from each animal and fixed in 10% formalin at a temperature of 40˚C for 2 days. The tissue was decalcified in EDTA (10%) and embedded in liquid paraffin. Tissue sections of 4-µm thickness were cut from the wax blocks using a microtome and then stained with haematoxylin and eosin (H&E) or used to determine TRAP activity in osteoclasts using the leukocyte acid phosphatase assay kit as recommended by the manufacturer.

### Statistical analyses

All data are expressed as the mean ± standard error of the mean (SEM, n=8). Data were analysed using one-way analysis of variance followed by post-hoc comparisons of the means using Dunnett’s post-hoc test in GraphPad Prism (ver. 6.1; San Diego, CA, USA). A P<0.05 was considered to indicate statistical significance.

## Results

### Fuscoside ameliorates the bone defect

**Fig.1:**
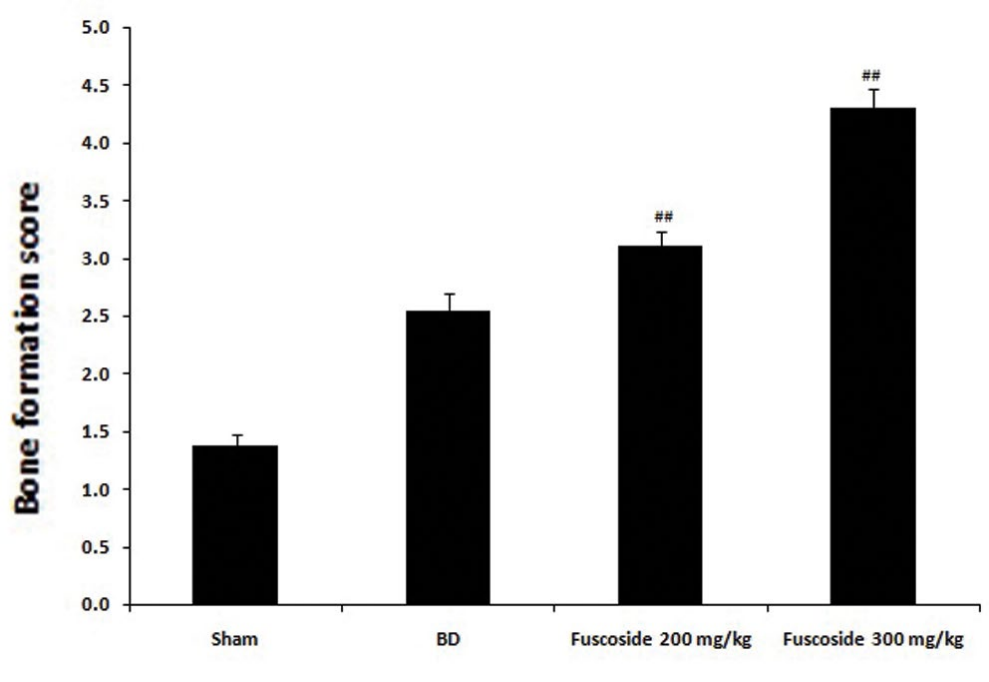
Bone formation scores of sham-operated, BD and fuscoside-treated BD rats. Data are presented as mean ± SEM (n=8). BD; Bone defect and ^##^; P< 0.01 vs. BD group.

The bone formation scores of the four groups of rats are shown in Figure 1. Bone formation increased in the BD group but was higher in the fuscoside 200 and 300 mg/kg groups (3.1 and 4.3, respectively).

### Fuscoside ameliorates the levels of biochemical markers in bone defect rats

The levels of several bone-related biochemical markers in the serum of rats from the four groups are shown in Table S2 (See Supplementary Online Information at www.celljournal.org). The serum OC level was lower in the BD group than in the sham-operated group (2.93 pg/mL vs. 9.47 pg/mL, respectively), while the serum CTX level was increased (152.9 ng/mL vs. 32.73 ng/mL, respectively), as was BSAP activity (15.33 U/L vs. 6.18 U/L, respectively). Fuscoside at both doses enhanced the serum level of OC to 7.84 pg/mL while reducing the CTX level to 61.29 ng/mL and BSAP activity to 8.72 U/L.

### Fuscoside ameliorates the level of cytokines in bone defect rats

**Fig.2:**
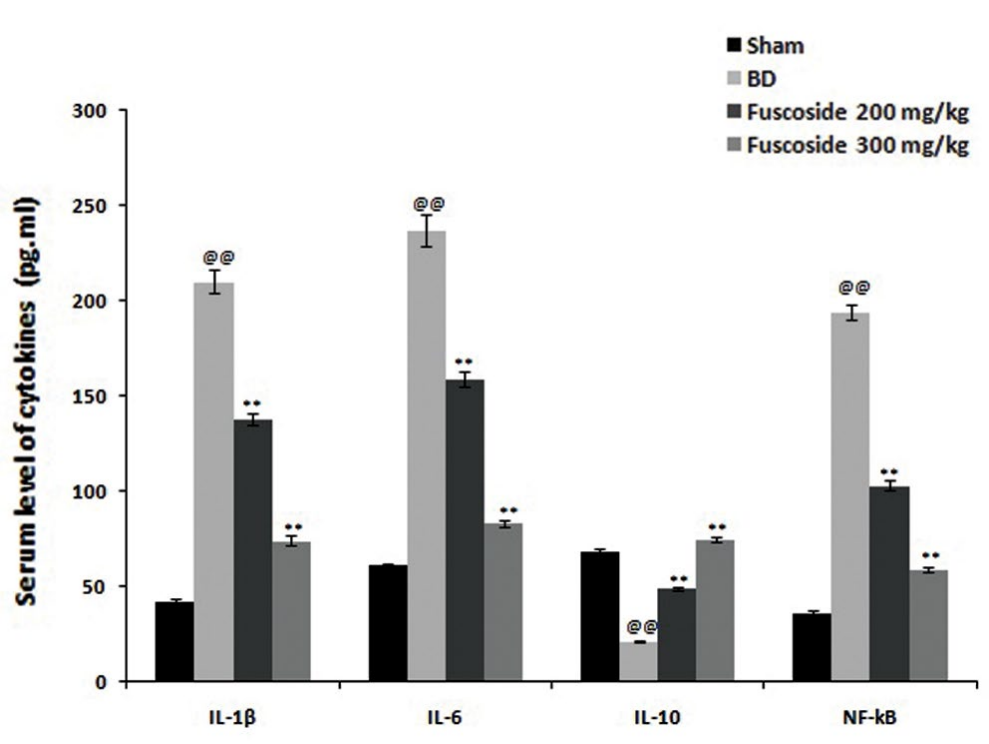
Levels of inflammatory cytokines in the serum of sham-operated, BD and fuscoside-treated BD rats. Data are presented as mean ± SEM (n=8). BD; Bone defect group, IL; Interleukin, NF-kB; Nuclear factor kappa-light-chain-enhancer of activated B cells, ^@@^; P<0.01 vs. sham-operated group, and **; P<0.01 vs. BD group.

The serum concentrations of the cytokines IL-1β, IL-6, IL-10 and NF-kB in the four groups of rats were also determined. IL-1β, IL-6 and NF-kB levels were enhanced, while the level of IL-10 was reduced, in the serum of BD versus sham-operated rats. However, comparison of the fuscoside-treated and BD groups showed a reduction in serum IL-1β, IL-6 and NF-kB levels and an increase in the level of IL-10 (Fig .2).

### Fuscoside intervenes in the Rankl/Nlrp3/Opg pathway

**Fig.3:**
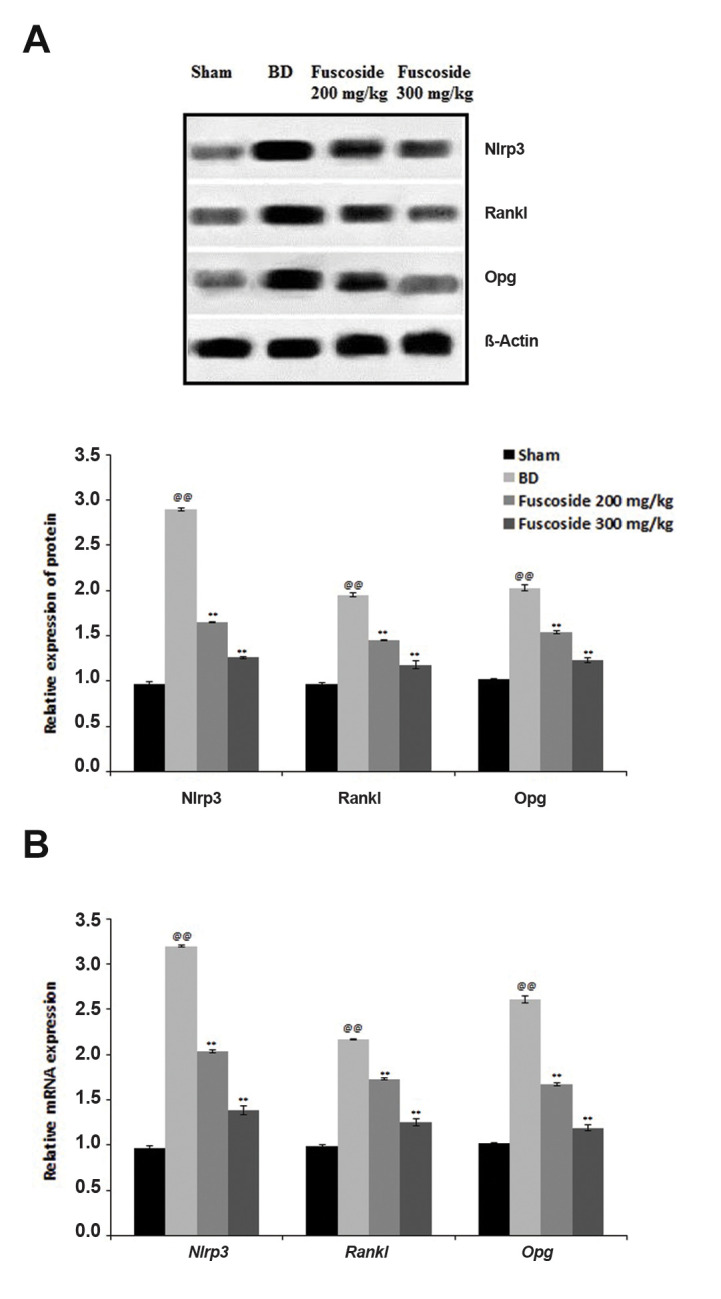
Effect of fuscoside on the Rankl/Nlrp3/Opg pathway in tibial tissue of BD rats. **A.** Expression of Nlrp3, Rankl and Opg protein as determined by western blot. **B. **mRNA expression of *Nlrp3, Rankl *and *Opg* as determined by qRT-PCR. Data are presented as mean ± SEM (n=8). BD; Bone defect group, qRT-PCR; Real-time quantitative reverse transcription polymerase chain reaction,^@@^; P<0.01 vs. sham-operated group, and **; P<0.01 vs. BD group.

The effect of fuscoside on Nlrp3, Rankl and Opg, expression in the tibial tissue of the four groups of rats was assessed by qRT-PCR and western blot (Fig .3A, B). This result was confirmed at the protein level, as the expression of Nlrp3, Rankl and Opg proteins was enhanced in the tibial tissue of BD versus sham-operated rats. The opposite results were obtained in fuscoside-treated versus BD rats, i.e. the expression of Nlrp3, Rankl and Opg was reduced (Fig .3A). The relative mRNA and protein expression of Nlrp3, Rankl and Opg increased in the BD group compared to the sham-operated group. However, fuscoside treatment altered Nlrp3, Rankl and Opg mRNA expression (Fig .3B).

### Fuscoside ameliorates the Runx2/Osterix/Oc axis

**Fig.4:**
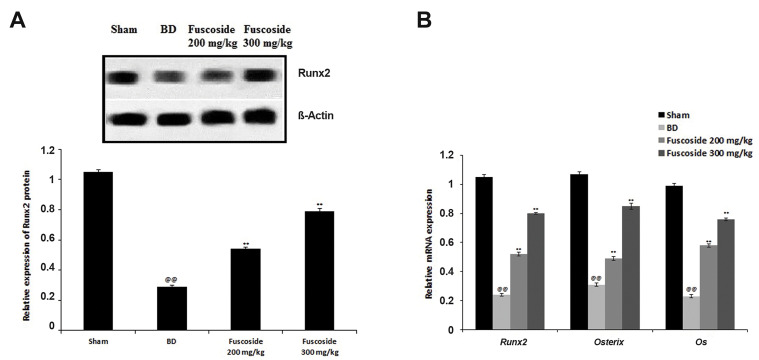
Effect of fuscoside on The Runx2/Osterix/Oc Axis in tibial tissue of BD rats. **A.** Expression of Runx2 protein as determined by western blot. **B. **mRNA expression of *Runx2, Osterix* and *Oc* as determined by qRT-PCR. Data are presented as mean ± SEM (n=8). BD; Bone defect group, qRT-PCR; Real-time quantitative reverse transcription polymerase chain reaction, ^@@^; P<0.01 vs. sham operated group, and **; P<0.01 vs. BD group.

The effect of fuscoside on the expression of Runx2, Osterix and Oc in the tibial tissue of the four groups of rats was assessed by qRT-PCR and western blot (Fig .4A, B). Data of the study confirmed that expression of Runx2 protein was reduced significantly in the BD group than fuscoside treated group. However, treatment of fuscoside was enhanced in the tibia tissue than BD group of rats (Fig .4A). The relative mRNA expression of *Runx2, Osterix* and *Oc* was reduced in the BD group compared to the sham-operated group. However, fuscoside treatment ameliorates the altered *Runx2, Osterix* and *Oc* mRNA expression (Fig .4B).

### Fuscoside ameliorates the apoptosis of osteocytes

**Fig.5:**
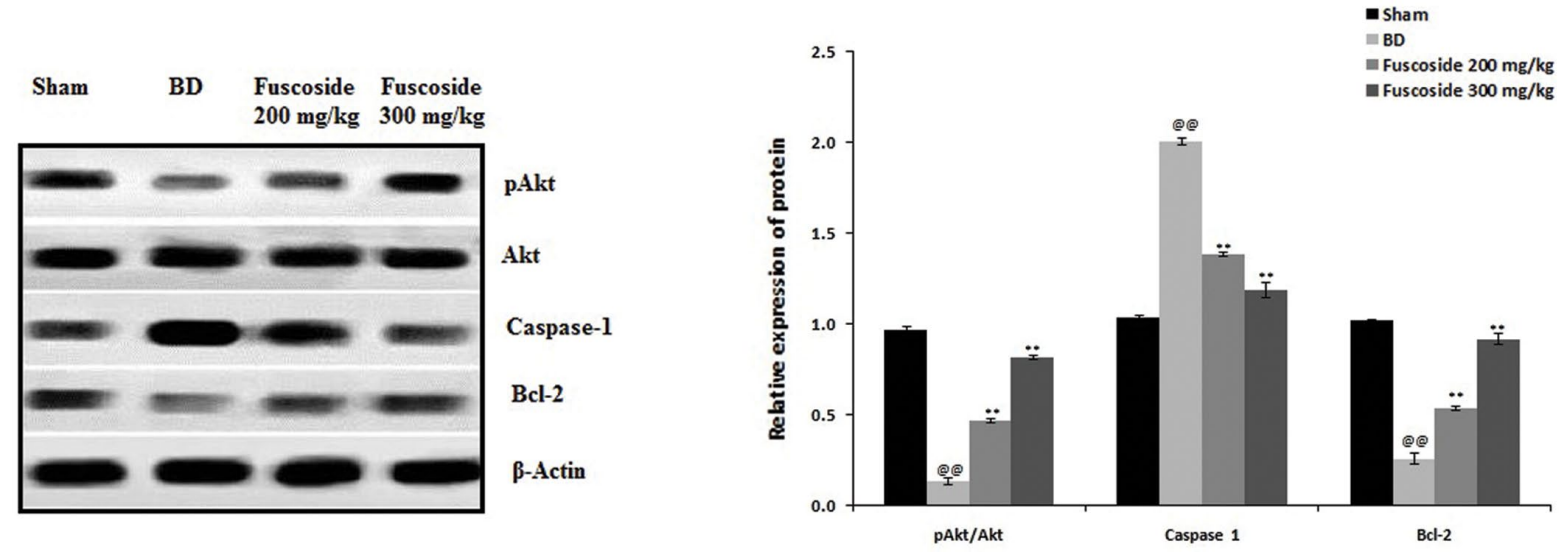
Effect of fuscoside on p-Akt, Akt, Caspase-1 and Bcl-2 expression in the tibial tissues of BD rats. Data are presented as mean ± SEM (n=8). BD; Bone defect group, ^@@^; P<0.01 vs. sham operated group, and **; P<0.01 vs. BD group.

Figure 5 shows the effect of fuscoside on the expression of apoptosis proteins in the tibial tissues of the four groups of rats. The p-Akt/Akt ratio and the level of Bcl-2 protein were lower, whereas the level of Caspase-1 was higher, in the BD rats than in the sham-operated rats. These effects were reversed by fuscoside treatment.

### Fuscoside ameliorates the histopathology of tibia tissue

**Fig.6:**
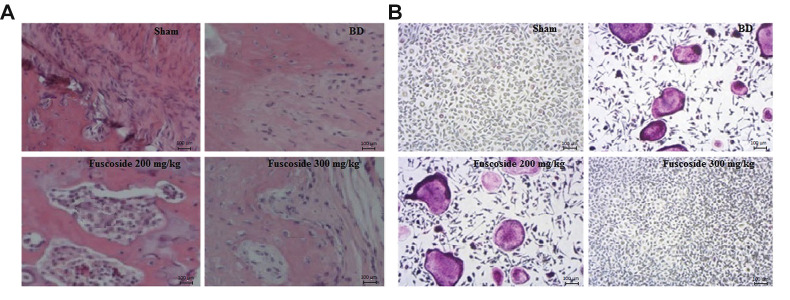
Histopathological changes in the tibial tissue of BD rats with fuscoside treatment. **A.** H&E staining and **B. **TRAP staining (scale bar: 100 µm). BD; Bone defect group.

Histopathological changes in the tibia of fuscoside-treated BD rats were observed by H&E and TRAP staining (Fig .6A, B). In the BD group, increased inflammatory infiltrate, including neutrophils, and irregular granulation tissue compared to the sham-operated group were seen. However, fuscoside treatment alleviated both changes at the bone remodelling site. Moreover, in the bone tissue of the BD group, the number of TRAP-positive multinucleated osteoclasts was higher than that in the sham-operated rats, whereas in the tissue of the fuscoside-treated group, the number of TRAP-positive multinucleated osteoclast was lower than that in the BD group (Fig .6B).

## Discussion

BDs occur due to the undesirable growth of bone tissue at the site of bone fracture and growth plate injury. However, there are very few drugs available for the management of a BD or for the proper formation of bone and those that have been tried have several limitations. This study demonstrated the beneficial effect of fuscoside on the prevention of BD formation and provided insights into the underlying molecular mechanism. The efficacy of fuscoside in sham-operated, BD, and fuscoside-treated rats was evaluated based on the bone formation score and the serum levels of cytokines and biochemical markers. Studies of the mechanism of action consisted of mRNA- and protein-based analyses of the Rankl/Nlrp3/Opg pathway in tibial tissue using qRT-PCR and western blotting. Histopathological changes were observed by comparing H&E and TRAP staining in the four groups of rats.

The development of a BD may be due to internal infection, trauma to the bone tissue or an inability of the body to heal properly, all of which affect correct bone formation (14). BD management is aimed at enhancing bone formation, which in this study was achieved by fuscoside treatment as evidenced by the improved bone formation scores in fuscoside-treated BD rats. In patients with a BD, the serum levels of OC, CTX and BSAP, which are markers of bone formation and osteocyte function, are altered (15). However, the serum levels of all three bone markers improved in the BD rats treated with fuscoside.

Recent studies have suggested a role for the Nlrp3 cascade in the relationship between inflammation and many diseases, including diabetes, bronchial asthma and atherosclerosis, but also in BD formation (16, 17). Nlrp3 activation enhances inflammatory cytokine concentrations (18) and the expression of apoptosis proteins such as Bcl-2 and Caspase-1. Our data showed that both the activation of Nlrp3, and thus of the apoptosis cascade, and the increase in IL levels were reversed in BD rats following fuscoside treatment.

Bone repair and healing includes the differentiation and proliferation of osteogenic cells, mediated by biological factors such as Runx2 and Oc (19, 20). Treatment with fuscoside targets this sequence of events, as demonstrated in our study. In the development of a BD, the differentiation of osteoclasts is enhanced and that of osteoblasts is reduced (21). In osteoclasts, differentiation involves the activation of osteoclastogenic Rankl and the deactivation of Opg, an inhibitor of Rankl expression (22, 23). In the fuscoside-treated BD rats, the altered expression Rankl/ Opg in the tibial tissue of the BD rats was attenuated. The ability of fuscoside to reverse the pathological changes in the bone tissue of BD rats was further demonstrated histopathologically. 

## Conclusion

This study demonstrated the protective effect of fuscoside in BD formation via a mechanism that included reductions in osteoclast differentiation and inflammatory cytokine levels, as well as the regulation of the Rankl/ Nlrp3/Opg pathway.

## Supplementary PDF


