# Supplementary material for: Fuscoside Attenuates Bone Loss in Bone Defects by Regulating The Rankl/Nlrp3/Opg Pathway in Rats
Source: Cell J. 2021 Aug 29;23(4):451–6. doi: 10.22074/cellj.2021.7736 (PMC8405081; doi:10.22074/cellj.2021.7736)
Supplement: Supplementary file 1 [file Cell-J-23-451-s01.pdf]

CELL JOURNAL (Yakhteh)

Supplementary Information for

Fuscoside Attenuates Bone Loss in Bone Defects by Regulating The Rankl/Nlrp3/Opg Pathway in Rats

Xiangwei Liu, M.D., Binfeng Wang, M.D.*

Department of Traumatic Orthopedics, Chifeng Municipal Hospital, Chifeng, Inner Mongolia, China

***Corresponding Address: Department of Traumatic Orthopedics, Chifeng Municipal Hospital, Chifeng, Inner Mongolia, China**
Email: wangbinfeng1258@qq.com

Table S1: Gene sequence of primers

Sr. No.	Gene	Primer sequence (5´-3´)
1	*Rankl*	F: CCGTGCAAAGGGAATTACAAC<br>R: GAGCCACGAACCTTCCATCA
2	*Nlrp3*	F: GGACTGAAGCACCTGTTGTGCA<br>R: TCCTGAGTCTCCCAAGGCATTC
3	*Opg*	F: GGAGACACAGCTCGCAAGA<br>R: GCACACTGCTTTCACAGAGG
4	*Runx2*	F: TCACAAATCCTCCCCAAGTGG<br>R: GAATGCGCCCTAAATCACTGA
5	*Osterix*	F: GCTTTTCTGTGGCAAGAGGTTC<br>R: CTGATGTTTGCTCAAGTGGTCG
6	*Oc*	F: GCTGGCCCTGACTGCATTCTG<br>R: ATTCACCACCTTACTGCCCTCCTG
7	*β-Actin*	F: CACCATTGGCAATGAGCGGTTC<br>R: AGGTCTTTGCGGATGTCCACGT

Table S2: Influence of fuscoside on serum biochemical markers in bone defect rats

Group	OC (pg/mL)	CTX (ng/mL)	BSAP (U/L)
Sham	9.47 ± 037	32.73 ± 3.18	6.18 ± 0.27
BD	2.93 ± 0.28##	152.9 ± 8.06##	15.33 ± 0.63##
Fuscoside 200 mg/kg	5.61 ± 0.51**	106.73 ± 5.39**	12.15 ± 0.41**
Fuscoside 300 mg/kg	7.84 ± 0.47**	61.29 ± 2.74**	8.72 ± 0.25**

Data are presented as mean ± SEM (n=8). BD; Bone defect group, OC; Osteocalcin, CTX; C-terminal telopeptide of type 1 collagen, BSAP; Bone specific alkaline phosphatase, ##; P<0.01 vs. sham-operated group, and **; P<0.01 vs. BD group.
